# Urinary carbonic anhydrase 1 as a marker of hematuria in IgA nephropathy

**DOI:** 10.1093/ndt/gfaf133

**Published:** 2025-07-15

**Authors:** Tomas G J M Post, Nils Rother, Elmar Pieterse, Ilse M Rood, Luuk B Hilbrands, Jonathan Barratt, Raphaël Duivenvoorden

**Affiliations:** Department of Nephrology, Research Institute for Medical Innovation, Radboud University Medical Center, Nijmegen, The Netherlands; Department of Nephrology, Research Institute for Medical Innovation, Radboud University Medical Center, Nijmegen, The Netherlands; Department of Nephrology, Research Institute for Medical Innovation, Radboud University Medical Center, Nijmegen, The Netherlands; Department of Nephrology, Research Institute for Medical Innovation, Radboud University Medical Center, Nijmegen, The Netherlands; Department of Nephrology, Research Institute for Medical Innovation, Radboud University Medical Center, Nijmegen, The Netherlands; Department of Cardiovascular Sciences, University of Leicester, Leicester, UK; Department of Nephrology, Research Institute for Medical Innovation, Radboud University Medical Center, Nijmegen, The Netherlands; Biomolecular Engineering and Imaging Institute, Icahn School of Medicine at Mount Sinai, New York, NY, USA

To the Editor,

In immunoglobulin A nephropathy (IgAN) hematuria is a risk factor for the development of kidney failure and a decrease in hematuria is associated with improved kidney survival [1]. Hematuria can be quantified by (automated) conventional bright field microscopy of the urinary sediment. However, because erythrocytes lyse rapidly in urine, it is difficult to reliably measure hematuria in urine samples that are collected and centrally stored in large multicenter studies. As a result, treatment effects on hematuria are often not reported in clinical trials. Additionally, it has been reported that automated microscopic analysis underestimates the severity of hematuria in kidney diseases [2–4]. Therefore, there is a need for a quantitative marker of hematuria that can be measured in a standardized manner in stored urine samples. We investigated whether carbonic anhydrase 1 (CA1) is a useful marker of hematuria that can serve as an alternative to microscopic erythrocyte quantification in urine. Carbonic anhydrases (CA) are zinc-metallo enzymes, responsible for catalyzing the reversible hydration of carbon dioxide, of which 12 isoforms exist. CA1 is primarily located in the cytosol of red blood cells and is the most abundant protein in erythrocytes, besides hemoglobin [5, 6].

To investigate the value of CA1 as a marker of hematuria, we first performed an *in vitro* experiment using blood and urine from a healthy subject (Fig. 1a; for full methods see Supplementary Material). Erythrocytes were isolated and diluted in clean urine to concentrations reflecting the clinically observable range of 5–1000 erythrocytes per high-power field (hpf) in the urine sediment. Samples were left at room temperature for different periods of time, and part of the urine was centrifuged prior to freezing. After thawing the urine samples the CA1 concentration was determined using an enzyme-linked immunosorbent assay (ELISA). We found a logarithmic relationship between the concentration of CA1 and the number of erythrocytes added to the urine (Fig. 1b). After centrifugation, low levels of CA1 could still be measured, most likely due to lysis of erythrocytes prior to centrifugation. The CA1 levels after centrifugation also had a logarithmic relationship with the number of added erythrocytes. After demonstrating that urine CA1 levels correlated with urine erythrocyte numbers, we investigated the correlation between urine CA1 levels and hematuria as quantified by automated microscopy of urinary sediments in a cohort of 41 IgAN patients from the Radboudumc in Nijmegen, the Netherlands, and 35 healthy controls. Patients had a mean age of 39 years [standard deviation (SD) 14.5 years], a mean body mass index (BMI) of 24.9 kg/m^2^ (SD 4.2 kg/m^2^), a mean estimated glomerular filtration rate (eGFR) of 59.9 mL/min/1.73 m^2^ (SD 26.6 mL/min/1.73 m^2^) and a mean protein/creatinine ratio of 1.04 mg/mg (SD 0.93 mg/mg), 30% were female, 90% were treated with renin–angiotensin–aldosterone system (RAAS) inhibitors and none had previously been treated with immunosuppressive medication. Erythrocyte counts in the urinary sediment, determined by automated microscopy, and CA1 levels in the urine were higher in IgAN patients than in the healthy controls (Fig. 1c). In a multivariate linear regression analysis with backward elimination we found no association between the urinary CA1 level and age, BMI, eGFR, proteinuria, sex or RAAS inhibitor use. We found no association between the MEST-C scores and erythrocyte count or urinary CA1 level in a subgroup of 10 patients that had a kidney biopsy within 3 years before study inclusion. The urinary CA1 levels showed a high correlation with erythrocyte counts, with a Spearman's rho of 0.459 (*P* < .001; Fig. 1d) and an interclass correlation coefficient of 0.702. CA1 levels and erythrocyte counts showed the strongest correlation in urine sediments with >25 erythrocytes per high-power field (hpf). The weaker correlation between CA1 levels and erythrocyte counts in urine sediments with <25 erythrocytes per hpf could be the result of the decreased reliability of automated microscopy analysis at lower levels of hematuria [2]. Multivariable regression analysis showed that the number of erythrocytes determines CA1 levels (*P* < .001) independent of the protein concentration in the urine (g/L), kidney function (eGFR Chronic Kidney Disease Epidemiology Collaboration, mL/min/1.73 m^2^), age and sex (Supplementary data, Table S1). To investigate urinary CA1’s capacity to identify IgAN patients with hematuria (erythrocytes >5/hpf), we performed a receiver operating characteristic (ROC) curve analysis, yielding an area under the curve (AUC) of 0.734 (Fig. 1e). Discriminative performance further improved for identification of IgAN patients with erythrocytes >25/hpf (AUC of 0.886) and erythrocytes >50/hpf (AUC of 0.936), which are erythrocyte counts that are usually observed in patients with active IgAN.

**Figure 1:**
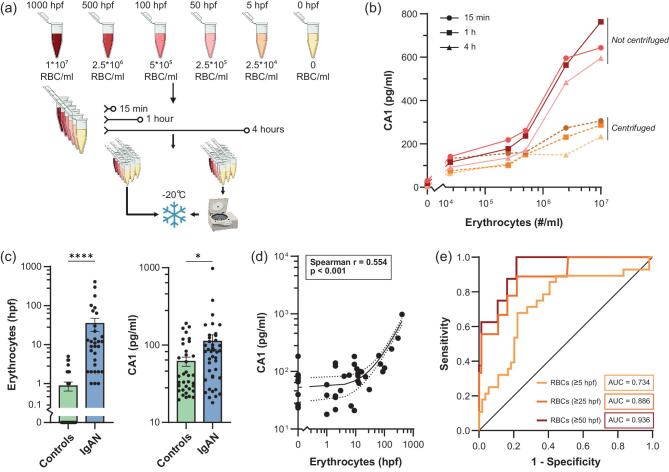
CA1 concentrations correlate with the number of erythrocytes in urine (**a**) Experimental set up to determine the relation between CA1 and erythrocytes in urine. Clean urine was spiked with different concentrations of red blood cells and incubated at room temperature for either 15 min, 1 h or 4 h. Half of the urine was centrifuged before storing the supernatant at –20°C, the other half was stored directly. Measured erythrocyte concentrations and the reflected clinically observable range of 5–1000 erythrocytes per hpf are shown. (**b**) Relationship between concentration of erythrocytes (#/mL) and concentration of CA1 (pg/mL) in urine. (**c**) Erythrocyte count (#/hpf) in urinary sediment and CA1 concentration (pg/mL) differ significantly between IgAN patients (*n* = 41) and healthy controls (*n* = 35) (*P* < .0001 and *P* < .05, respectively). Bar charts with mean and standard error of the mean. (**d**) Spearman correlation between erythrocyte count in urinary sediment and CA1 in the urine of 41 IgAN patients. (**e**) ROC curves for the detection of IgAN patients with hematuria, with erythrocyte counts of ≥5, ≥25 or ≥50 per hpf. RBC, red blood cells.

Our data reveal urinary CA1 as a biomarker for hematuria, which warrants further investigation in other IgAN patient cohorts for validation. Our data demonstrate that urinary CA1 can be used as a marker of hematuria. No reliable assays currently exist to determine hematuria in stored urine samples, as erythrocytes lyse rapidly in urine, and average hemoglobin dipsticks provide only qualitative information on hematuria. The commercially available CA1 ELISA assay is a test that can be performed in a standardized manner and offers the ability to estimate hematuria from frozen stored urine samples, and is independent of age, sex, proteinuria and kidney function. Our data suggest that the use of CA1 as a biomarker for hematuria can enable centralized assessment of pharmacologic effects on hematuria through the analysis of frozen urine samples from patients with IgAN (and possibly other glomerular diseases), collected in multicenter clinical trials.

## Supplementary Material

gfaf133_Supplemental_File
