# Supplementary material for: Urinary carbonic anhydrase 1 as a marker of hematuria in IgA nephropathy
Source: Nephrol Dial Transplant. 2025 Jul 15;40(11):2213–5. doi: 10.1093/ndt/gfaf133 (PMC12620611; doi:10.1093/ndt/gfaf133)
Supplement: gfaf133_Supplemental_File [file gfaf133_Supplemental_File.docx]

Urinary carbonic anhydrase 1 as a marker of hematuria in IgA nephropathy patients

**Supplementary Material**

**Supplementary Methods** p. 1-2
 *Study design and participants
 In vitro experiment
 Erythrocyte quantification
 CA1 measurement
 Statistical analysis*

**Supplementary Results** p. 3
 *Table S1*

**Supplementary Methods**

***Study design and participants***

In this study the concentration of CA1 as a measure for hematuria was investigated both *in vitro* and in subjects with IgAN. The study was conducted at the Radboud university medical center in Nijmegen, The Netherlands from October 2021 to December 2022. All participants gave written informed consent. This study was approved by the Radboudumc Medical Review Ethics Committee (2021-13006) and adhered to the declaration of Helsinki.

IgAN patients (n=41) were enrolled in the study if they were between 18 to 70 years of age, had an estimated glomerular filtration ratio (eGFR CKD EPI) of 15 ml/min/1.73m2 or higher, and had a biopsy proven diagnosis. Controls were included if they were between 18 to 70 years of age and healthy. The control group consisted of both healthy individuals with no erythrocytes in the urine (n=23) and individuals with 1-5 erythrocytes per hpf in the urinary sediment (n=12). Patients were excluded if the IgAN coincided with or was considered to be secondary to another illness (e.g. liver disease, inflammatory bowel disease, celiac disease, skin disease, pulmonary disease). Exclusion criteria for cases and controls were presence of malignancy, current infection and/or antibiotic treatment in the past three months, current use of immunosuppressive medication in the past three months, pregnancy, an eGFR CKD EPI < 15 ml/min/1.73m2, being on dialysis or previously receiving a kidney transplantation, or inability to provide informed consent. IgA nephropathy patients were recruited by the treating physician at the outpatient clinic of the Radboudumc or by referral from other hospitals in the Netherlands. Controls were recruited by advertisement. Urine was collected and stored directly at -80°C for CA1 measurement.

***In vitro experiment***Erythrocytes were isolated from the blood of a healthy donor as described [4] and were diluted to concentrations ranging from 2.5*10^4^ to 10*10^6^ cells/ml, reflecting the clinically observable range of 5–1000 erythrocytes per high-power field (hpf) with microscopic analysis of the urinary sediment. Two series of clean urine were spiked with erythrocytes to obtain the dilutions mentioned above, and left at room temperature for 15 min, 1 hour, or 4 hours before freezing urine, with one series undergoing centrifugation for 5 min at 300 g prior to freezing. Urine samples were kept frozen at -20 °C for 7 days before CA1 measurement.

***Erythrocyte quantification***
Erythrocytes in the urine were quantified by automated microscopy of urinary sediments using the Roche Cobas U6500 U701 analyzer.

***CA1 measurement***
CA1 enzyme-linked immunosorbent assays (ELISA) were performed according to the manufacturer’s instructions using the Cusabi (CSB-EL004364HU) CA1 ELISA kit. Urine samples from patients and controls were not centrifuged after thawing prior to CA1 measurement.

***Statistical analysis***Statistical analyses were performed in IBM SPSS Statistics 29 and GraphPad Prism 10.2.2. Graphs were made using GraphPad Prism 10.2.2. Figures were made using Adobe llustrator 2024. Data are presented as mean ± SEM if not specified otherwise. Group sizes are indicated in the figure legends. Groups were assessed for normality, and compared using a Mann-Whitney U test.

**Supplementary Results**

**Table S1 | Multivariate linear regression analysis of clinical parameters and urinary CA1 concentration**

| **Characteristics** | **B** | **p-value** |
| --- | --- | --- |
| Erythrocytes (#/hpf) | 1.763 | < 0.001 |
| Proteinuria (g/l) | -8.403 | 0.525 |
| Kidney function (eGFR CKD-EPI) | -0.049 | 0.903 |
| Age (years) | 0.715 | 0.521 |
| Sex | -23.206 | 0.488 |

Dependent variable: CA1 (pg/ml).
